# An Ovarian Cancer Susceptible Gene Prediction Method Based on Deep Learning Methods

**DOI:** 10.3389/fcell.2021.730475

**Published:** 2021-08-13

**Authors:** Lu Ye, Yi Zhang, Xinying Yang, Fei Shen, Bo Xu

**Affiliations:** ^1^Department of Gynecology, Guangdong Second Provincial General Hospital, Guangzhou, China; ^2^Department of Thyroid Surgery, Guangzhou First People’s Hospital, School of Medicine, South China University of Technology, Guangzhou, China

**Keywords:** ovarian cancer, gene prediction, omics data, deep learning method, pathway analysis

## Abstract

Ovarian cancer (OC) is one of the most fatal diseases among women all around the world. It is highly lethal because it is usually diagnosed at an advanced stage which may reduce the survival rate greatly. Even though most of the patients are treated timely and effectively, the survival rate is still low due to the high recurrence rate of OC. With a large number of genome-wide association analysis (GWAS)-discovered risk regions of OC, expression quantitative trait locus (eQTL) analyses can explore candidate susceptible genes based on these risk loci. However, a large number of OC-related genes remain unknown. In this study, we proposed a novel gene prediction method based on different omics data and deep learning methods to identify OC causal genes. We first employed graph attention network (GAT) to obtain a compact gene feature representation, then a deep neural network (DNN) is utilized to predict OC-related genes. As a result, our model achieved a high AUC of 0.761 and AUPR of 0.788, which proved the accuracy and effectiveness of our proposed method. At last, we conducted a gene-set enrichment analysis to further explore the mechanism of OC. Finally, we predicted 245 novel OC causal genes and 10 top related KEGG pathways.

## Introduction

Ovarian cancer (OC) is one of the major lethal diseases for women, despite ranking tenth in morbidity rate, it is the fifth leading cause of death among cancers (Siegel et al., 2011). Usually, OC is diagnosed at an advanced stage which induced a high death rate. However, even patients got primary treatment such as surgical resection and adjuvant drug therapy, the high rate of recurrence, and high incidence of advanced stage disease eventually caused a high mortality rate (Armstrong, 2002). In terms of treating OC and reducing the high fatality rate to improve survival in OC patients, studies have been exploring the development of new treatment, and effective chemotherapies (Badgwell and Bast, 2007; Kobayashi et al., 2012). While efforts and contributions have been made to improve the treatment, cure rates have not been raised significantly. Thus, it is more important to explore the mechanism and underlying biological pathogenic factors of OC to better understand the disease, and find a better treatment.

Genome-wide association analysis (GWAS) have identified hundreds of risk genetic variants (SNPs) associated with OC (Song et al., 2009; Bolton et al., 2010; Goode et al., 2010; Permuth-Wey et al., 2013; Pharoah et al., 2013; Shen et al., 2013). However, they can only explain a small fraction of disease risk regions in a functional way (Pomerantz et al., 2010; Grisanzio et al., 2012; Bojesen et al., 2013). It is widely known that most of the risk alleles are located in the nonprotein coding regions of the genome, indicating that most of them are functional regulators of the expression of target genes (Hazelett et al., 2014). Thus, it is not comprehensive to identify disease-related genes by merely being dependent on GWAS datasets. To provide additional evidence for exploring risk genes, expression quantitative trait locus (eQTL) analysis is a direct method to explore candidate genes at risk loci. Since most transcripts are regulated by genes, eQTL can identify genetic variants related to the expression level of genes. eQTL analyses have identified multiple causal genes for different cancer types such as prostate, breast, colorectal, and kidney cancers (Loo et al., 2017; Yang et al., 2017; Beesley et al., 2020; Bicak et al., 2020). Therefore, it is more creditable to identify OC-related genes based on the combination of GWAS and eQTL data.

Besides, over the past decades, numerous noncoding RNAs (ncRNAs), such as lncRNA, siRNA, piRNA, and miRNA have been detected to execute the regulation function by interacting with target genes (Esteller, 2011; Lee and Young, 2013). In humans, it is estimated that the number of ncRNA genes are more than twice as many as that of protein-coding genes (Bunch et al., 2016). Thus, ncRNAs have been considered key regulators of multiple biological processes and development. Along with the rapid advancement of high-throughput sequence analyses of ncRNAs, more and more transcriptional mechanisms have been illustrated. ncRNAs should also be regarded as a major factor to explore the pathologies of OC due to its regulation function of gene expression. Hence, it is important to take into consideration the role of regulatory ncRNAs to identify OC-related causal genes.

However, along with the rapid development of understanding the mechanisms of complex disease, there are a few computational methods to predict disease genes based on various gene features. In this study, we aimed to identify susceptibility genes associated with OC based on integrated gene features. We first employed graph attention network (GAT) to learn the compact gene feature from a gene interaction network with gene features, then employed a deep neural network (DNN) to predict OC-related susceptibility genes. To further explore the mechanism of OC, we also performed a gene-set enrichment analysis to predict more related pathways in OC process.

## Materials and Methods

### Work Frame

**FIGURE 1:**
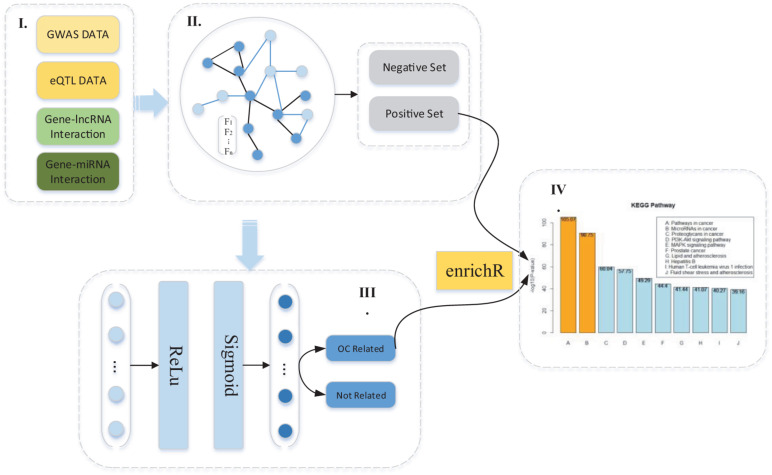
The pipeline of ovarian cancer (OC) causal gene prediction method.

In this study, our method contains four main parts, feature extraction, compact gene feature learning based on GAT, and OC-related susceptibility gene prediction based on DNN and OC-related pathway analysis. In the first section, we extracted gene features based on integrated GWAS data, eQTL data, and published data of gene-related lncRNAs and miRNAs. In total, we extracted a 2,664-dimensional feature representation from four gene features. We then utilized a graph neural network with attention mechanism (GAT) to learn the compact gene feature for a low-dimension feature representation in order to obtain a better classification performance in the prediction process. The low-dimension feature matrix is considered the input of DNN to train the model and conduct the prediction process. After obtaining the predicted causal genes related to OC, we further performed a pathway analysis based on enrichR (Chen et al., 2013; Kuleshov et al., 2016), and a gene-set enrichment tool to find more related kyoto encyclopedia of genes and genomes (KEGG) pathways for a better understanding of the mechanism of OC. The workflow is presented in Figure 1.

### Data Collection

We first downloaded and verified 3,181 OC-related genes from DisGeNET database (Piñero et al., 2017, 2020) as a positive dataset. To build a gene-gene interaction network, we downloaded gene interaction information from HumanNet database (Hwang et al., 2019). For constructing a balanced training set, we randomly selected 3,171 genes which have interactions with positive genes from HumanNet as a negative set. To extract ncRNA-gene interaction feature, we downloaded gene-lncRNA association and gene-miRNA association information from LncRNA2Target, and miRTarBase, respectively (Hsu et al., 2011; Jiang et al., 2015; Cheng et al., 2019; Huang et al., 2020). MiRTarBase is a database providing comprehensive information based on experimentally verified miRNA-target interactions curated from published articles; it accumulated over 13,404 validated associations. LncRNA2Target is a database storing comprehensive lncRNA-target interactions inferred from published articles and experiments.

The GWAS data providing OC susceptibility loci was downloaded from GWAS catalog database, accession ID GCST90011821 (MacArthur et al., 2017). They sampled from 1,259 European ancestry cases and 410,350 controls providing genetic variant loci related to OC. eQTL data in ovary tissue was downloaded from GTEx database (Lonsdale et al., 2013). Finally, our training set is constructed based on 3,181 positive genes and 3,171 negative genes for further feature extraction.

### Feature Extraction

We extracted gene features from four aspects, susceptibility loci derived from GWAS, eQTL data from ovary tissue, interactions between genes, and miRNAs/lncRNAs. We first obtained the detail location information of training genes containing chromosome name, start position, and end position. Then, we mapped the genes to the susceptibility loci and sorted by *p*-value provided by GWAS data. We extracted the top five significant *p*-values as GWAS feature of the gene. Thus, the GWAS feature of a gene can be represented as a 5-D vector:

(1) $$F_{\text{GWAS}}=\left[D_{1},D_{2},D_{3},D_{4},D_{5}\right]$$

For those genes that cannot be mapped to five SNPs, the feature value is set to one. To extract eQTL-based gene feature, we mapped the genes to eQTL data based on gene location information, and extracted the top five significant *p*-values as eQTL feature, set feature value to one if a gene cannot map to five SNPs. Thus, the eQTL feature can also be represented as a 5-D vector:

(2) $$F_{\text{eQTL}}=\left[D_{1},D_{2},D_{3},D_{4},D_{5}\right]$$

From the gene-lncRNA interaction obtained from lncRNA2Target, we filtered the interactions to make sure each of the training genes is related to at least one lncRNA. As a result, 59 lncRNAs are preserved. Thus, the lncRNA feature of a gene can be denoted as a 59-D vector, where the value is 1 if the gene is related to lncRNA[*i*], the value is set to 0 vice versa.

(3) $$F_{\text{lncRNA}}=\left[D_{1},D_{2},D_{3},,D_{59}\right]$$

(4) $$D_{i}=\left\{ \begin{array}{ccc} 0, & i\,f & g\,e\,n\,e\,i\,s\,n\,o\,t\,r\,e\,l\,a\,t\,e\,d\,t\,o\,l\,n\,R\,N\,A_{i}\\ 1.\; & i\,f & g\,e\,n\,e\,i\,s\,r\,e\,l\,a\,t\,e\,d\,t\,o\,l\,n\,R\,N\,A_{i} \end{array}\right.$$

We then filtered the gene-miRNA interactions to make sure each of the training genes is related to at least one miRNA. As a result, 2,595 miRNAs are preserved. Thus, the miRNA feature of a gene can be denoted as a 2,595-D vector:

(5) $$F_{\text{miRNA}}=\left[D_{1},D_{2},D_{3},,D_{2595}\right]$$

(6) $$D_{i}=\left\{ \begin{array}{ccc} 0, & i\,f & g\,e\,n\,e\,i\,s\,n\,o\,t\,r\,e\,l\,a\,t\,e\,d\,t\,o\,m\,i\,R\,N\,A_{i}\\ 1.\; & i\,f & g\,e\,n\,e\,i\,s\,r\,e\,l\,a\,t\,e\,d\,t\,o\,m\,i\,R\,N\,A_{i} \end{array}\right.$$

Therefore, the feature representation of each gene in training set can be denoted as a 2,664 dimensional vector. Since the feature vector could be very sparse, we need to learn the compact feature representation to obtain a better classification performance.

### Compact Feature Learning Based on GAT

Sparse matrix is a matrix composed of mostly zero values, which often induces a poor classification performance in machine learning methods. Thus, we need to reconstruct the gene feature to get a low-dimensional feature representation. Since we can build a gene-gene interaction network based on gene association information obtained from HumanNet. A 6358^∗^6358 dimensional adjacent matrix can be constructed based on the network. Besides, each gene of the Internet also has a feature representation of itself. Thus, the gene network with gene features can be regarded as a graph-structured data; to make the gene prediction method more general, and we utilized a GAT as a feature learning model. GAT addressed the shortcomings of requiring costly matrix operation and dependency on preknowledge of graph structure by stacking layers in which nodes are participant in features of neighborhoods, and arranging attention weights to each nodes. Consider the node features being denoted as: $h=\left\{\vec{h_{\text{1}}},\vec{h_{\text{2}}},,\vec{h_{N}}\right\},\vec{h_{i}}\in {\mathbb{R}}^{F}$, where *N* is the size of training set and *F* is the dimension of gene features. The output of graph attentional layer is a new set of node features (of a low-dimension *F*’), denoted as: h′={h1′⇀′,h2′⇀′,,hN′⇀′},hi′⇀′∈ℝF′.

We then performed a self-attention on each node with a shared attentional mechanism a:*ℝ*^*F*^′ × *ℝ*^*F*^′ to compute attention coefficients:

(7) ei⁢j=a⁢(W⁢hi⇀i,W⁢hj⇀j)

where, *W* ∈ *ℝ*^*F*′*F*^ is a weight matrix applied to each node. *e*_*ij*_ denotes the importance of node *j* to node *i*. Based on this formulation, the model allows each node to participate with every other node and dropping structural information. For each node *j* in the neighborhood of node *i* (denoted as 𝒩_*i*_), we performed a softmax function to normalize the coefficients *e*_*ij*_:

(8) $$\alpha_{i\,j}=\text{softmax}\,\left(e_{i\,j}\right)=\frac{\exp\,\left(e_{\mathrm{ij}}\right)}{\sum_{k\in {𝒩}_{i}}\exp\,\left(e_{\mathrm{ik}}\right)}$$

After being activated by LeakyReLU function, *e*_*ij*_ can be denoted as:

(9) ei⁢j=LeakyReLU(a⇀[Whi⇀i,||Whj⇀j])

where, $\vec{a}$ ∈ *ℝ*^2*F*^′ is a weight vector; | | denotes the concatenation operation. Once obtained, the output feature of each node can be computed as a linear combination of the neighborhood node features with *e*_*ij*_:

(10) hi′⇀′=δ(∑j∈𝒩iαi⁢jWhj⇀j)

where, δ denotes a nonlinear transition. Thus, we obtained a low-dimension feature representation of the genes based on GAT.

### DNN Model Construction

Deep neural network has been regarded as a powerful tool in many domains of machine-learning applications. In this part, a binary-classification DNN model is used to predict OC-related genes based on the gene features derived from the output of GAT layer. The gene features were input to the DNN. The DNN model contains one hidden layer with a ReLU activation function and an output layer with a sigmoid activation function and a dropout technique. The sigmoid activation formulation is:

(11) $$\sigma \,\left(x\right)=1/\left(1+e^{-x}\right)$$

We used the Adam optimizer and binary cross-entropy function as the loss function. The loss function is:

(12) loss=-∑i=1ny′i⁢log⁡(yi)+(1-y′i)⁢log⁢(1-y′i)

### Training and Testing

To verify the performance of our GAT-DNN gene prediction model, we conducted a 10-fold cross-validation method on the training dataset containing 3,181 positive samples and 3,171 negative samples. The training set was randomly divided into 10 groups, nine of the 10 are regarded as training samples, and one left group is regarded as the test samples. The training samples were used to train the model and the last one was used to test the classification performance. This process was repeated 10 times to make sure the credibility of the verification.

## Results

### Measurement of Model Performance

**FIGURE 2:**
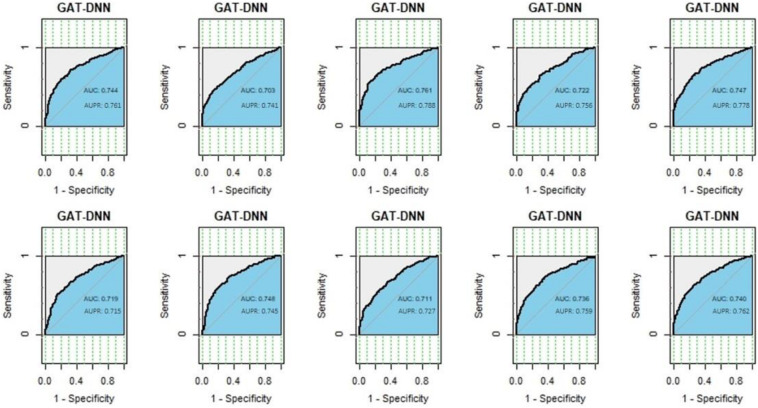
The performance of graph attention network-deep neural network (GAT-DNN) across a 10-fold cross-validations.

The performance of 10-fold cross-validation was assessed by area under curve (AUC) and the area under precision-recall curve (AUPR), as shown in Figure 2. As demonstrated in Figure 2, the AUC and AUPR are both over 0.7 across a 10-fold cross-validation, and which is a good performance for a classification model. The best performance is shown in the third validation with the AUC of 0.761 and the AUPR of 0.788, and which is chosen as the final prediction model to identify OC-related genes.

### Performance Comparison

**FIGURE 3:**
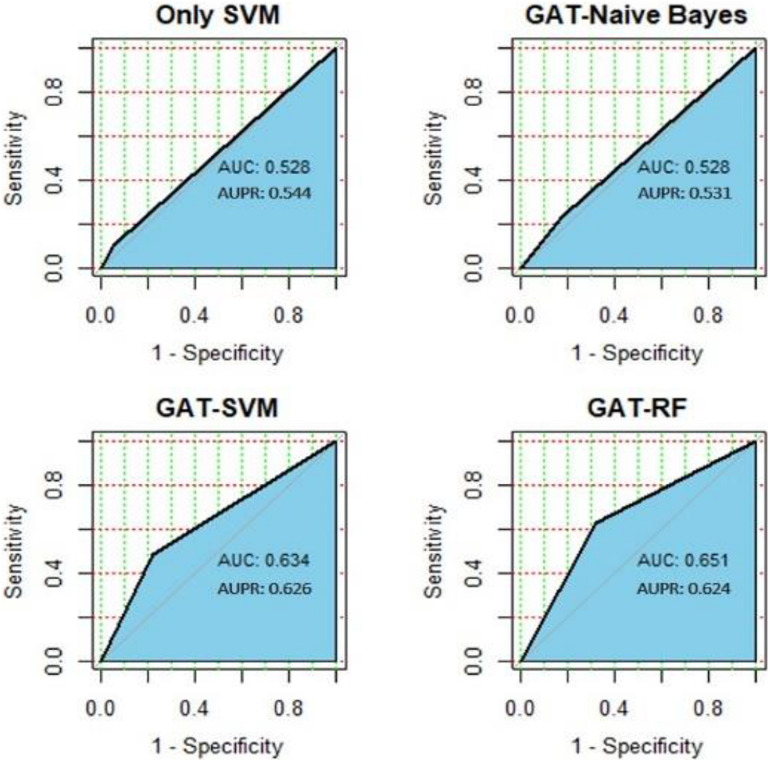
Results of method comparison.

To better illustrate the effectiveness and credibility of our method, we compared our model with other four combinations of machine learning methods with the same training set we used in model training part. We compared our model with GAT-SVM, only SVM (which means the gene features are not operated with GAT), GAT-Random Forest, and GAT-Naïve Bayes. The results are shown in Figure 3. As shown in the results, the performance is significantly poorer than our model. The best model is GAT-RF, with an AUC of 0.651 and AUPR of 0.624, which is approximately 0.1 lower than our GAT-DNN model. However, indicated from the performance of only SVM and GAT-SVM, it is obvious that the classification performance has been improved significantly after compact feature learning by GAT layer. Therefore, our model is the best to predict OC-related genes.

### OC Gene Prediction Process

Since we have demonstrated the effectiveness of our classification model based on a 10-fold cross-validation and comparison with other classification models, and we then performed the gene prediction process based on our built model on 721 ovary disease-related genes. These 721 candidate genes were downloaded from DisGeNET which is associated with ovary diseases but not OC. We extracted the gene features as mentioned in the section “Feature Extraction”. After compact feature learning by GAT layer, we reduced the dimension of gene features from 2,664 to 100. We then input the compact gene features to the DNN model; we finally predicted 245 of 721 candidate genes to be positively related to OC.

### Case Study

According to the results we obtained from gene prediction process, 245 of 721 candidate genes were predicted to be associated with OC. We listed the top 20 genes in Table 1. Backen et al. (2007) indicated that HS6ST1 are aberrant overexpression in carcinoma of ovary compared with normal ovaries. Natanzon et al. (2018) observed a significant association between methylation WDPCP expression in OC. KCNJ11 could be considered a favorable prognostic factor since they are observed to be expressed in OC according to the investigation of Fukushiro-Lopes et al. (2020). TBL2 was identified by Kim et al. (2012) as a DNA methylation regulated cancer antigen in OC. Park et al. (2014) investigated the expression of AGTR1 and AGTR2 in OC and revealed that the dual regulation of AGTR1/2 may be a therapeutic strategy since AGTR2 could antagonize the cancer cell proliferation induced by AGTR1.

**TABLE 1:** Top 20 predicted genes associated with ovarian cancer (OC).

Gene	Score	Gene	Score
HS6ST1	0.98354	TEAD2	0.88879
C2orf83	0.97384	PCSK1	0.88736
TM4SF4	0.95513	MTRR	0.88176
ARTN	0.95246	H6PD	0.87361
WDPCP	0.92387	EIF2B2	0.87341
KCNJ11	0.92073	SOX5	0.85771
TBL2	0.91197	NNMT	0.85176
AGTR2	0.90641	MIR324	0.84883
ATF1	0.90194	MIR33B	0.83557
PKD1	0.89846	TBPL2	0.83491

### Pathway Analysis

**FIGURE 4:**
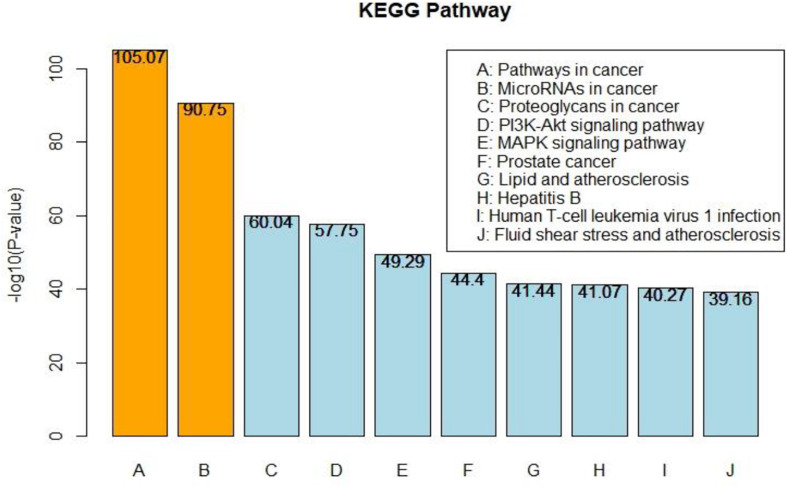
Pathway analysis based on OC-related genes.

After predicting the causal genes by our proposed model, we combined the published OC-related genes and our predicted genes with a total number of 3,426. We performed a pathway analysis on KEGG pathways using enrichr in order to further understand the mechanism of OC. Enrichr is a gene-set enrichment method to identify pathway enrichment among genes related to OC. The top 10 enriched pathways resulting from enrichr are shown in Figure 4. Enriched pathways are ordered by –log(*p*-value), obtained from a Fisher’s exact test.

Consistent with KEGG DISEASE database, top 2 OC-related pathways named Pathways in cancer (hsa05200) and MicroRNAs in cancer (hsa05206) are enriched among the predicted OC-related genes. Pathway proteoglycans (PGs) in cancer are known as a key pathway in understanding cancers since PGs in the tumor microenvironment are indicated to play important roles in contributing to biology of multiple types of cancer. The MAPK and PI3K-AKT pathway have been frequently observed to be important in OC, and both of the pathways are involved in OC cell migration (Jeong et al., 2013; Li et al., 2014). Understanding the pathways related to OC are important in revealing the underlying mechanism of OC.

## Discussion

In this article, we proposed an OC causal gene prediction method based on deep learning methods. We first extracted gene features considering ncRNA regulation function of gene expression and integrated GWAS and eQTL data. To learn a compact feature representation, we utilized a GAT, which can learn node features from a graph-structured data format without a preknowledge of the graph structure. After GAT layer, the feature dimension is reduced from 2,664 to 100. The new feature representations were then input to a DNN model which can learn gene features and perform a binary classification task. To demonstrate the performance of our proposed method, we conducted a 10-fold cross-validation on training set and made a comparison with other four integrated machine learning models. As a result, our model is significantly better than other models and achieved a high AUC of 0.761 and the AUPR of 0.788. We then employed the constructed model to predict causal genes and obtained 245 related genes. From the result of KEGG pathway analysis, we identified more OC-related pathways which are potential favorable evidence in understanding the mechanism of OC and provide new ideas for diagnosis and treatment.

## Data Availability Statement

The datasets presented in this study can be found in online repositories. The names of the repository/repositories and accession number(s) can be found in the article/Supplementary material.

## Ethics Statement

Ethical review and approval was not required for the study on human participants in accordance with the local legislation and institutional requirements. Written informed consent for participation was not required for this study in accordance with the national legislation and the institutional requirements.

## Author Contributions

LY, YZ, and BX participated in its design. LY, YZ, XY, and FS analyzed the data. LY, YZ, and FS wrote the manuscript. All authors read and approved the final manuscript.

## Conflict of Interest

The authors declare that the research was conducted in the absence of any commercial or financial relationships that could be construed as a potential conflict of interest.

## Publisher’s Note

All claims expressed in this article are solely those of the authors and do not necessarily represent those of their affiliated organizations, or those of the publisher, the editors and the reviewers. Any product that may be evaluated in this article, or claim that may be made by its manufacturer, is not guaranteed or endorsed by the publisher.
